# The Chemical and Products Database v4.0, an updated resource supporting chemical exposure evaluations

**DOI:** 10.1038/s41597-025-05240-0

**Published:** 2025-06-06

**Authors:** Sakshi Handa, Kristin K. Isaacs, Jonathan T. Wall, Allison Larger, Scott Burns, Lauren E. Koval, Kenta Baron-Furuyama, Colleen M. Elonen, David Lyons, Kathie L. Dionisio, M. Beth Horton, Katherine A. Phillips

**Affiliations:** 1 U.S. Environmental Protection Agency, Office of Research and Development, Center for Computational Toxicology and Exposure, Research Triangle Park, North Carolina USA; 2 General Dynamics Information Technology, Falls Church, Virginia USA; 3 Oak Ridge Associated Universities, Oak Ridge, Tennessee USA; 4 U.S. Environmental Protection Agency, Office of Research and Development, Center of Public Health and Environmental Assessment, Washington, District of Columbia USA; 5 U.S. Environmental Protection Agency, Office of Research and Development, Research Triangle Park, North Carolina USA

**Keywords:** Environmental sciences, Databases

## Abstract

Since the initial release of the Chemical and Products Database (CPDat) in 2018, the United States Environmental Protection Agency has added a considerable amount of chemical exposure-related information to the database and has expanded its schema to accommodate new types of data. This data descriptor provides information regarding the structure and types of data contained within CPDat (both existing and new), new controlled vocabularies implemented to harmonize terminology across the different data types, application of a rigorous data curation and quality assurance tracking system, and various methods of accessing CPDat.

## Background & Summary

Consumer products and other proximal (or “near-field”) sources have been identified as significant contributors to human exposure for many chemicals^1–3^. Quantitative data on the composition of consumer products is a key input for consumer exposure assessments that support chemical decision-making, including chemical screening and prioritization^4–7^. In addition, information on general chemical use is needed for informing decision-support tools and prioritization workflows. Chemical use information provides the foundation for the scoping phase of risk evaluations under the United States’ Toxic Substances Control Act (TSCA) by identifying “conditions of use” as required under the statute. Chemical use data can also inform a variety of risk-based prioritization activities under state programs within the United States^8,9^. Over the past decade, the Office of Research and Development (ORD) of the United States Environmental Protection Agency (EPA) has been aggregating general chemical use information with data on chemical ingredients in consumer products into the Chemical and Products Database (CPDat) to facilitate exposure assessment^10–12^. However, ongoing development and evolution of CPDat revealed a need for a better harmonized and curated database integrated with standardized quality assurance procedures. These procedures should be transparent, reproducible, efficient, and sustainable for use within EPA and stakeholder decision making.

To support the development of robust chemical use information for use in decision making, EPA has developed an internal, web-based data management and curation application, called Factotum, which facilitates the rapid collection and distribution of high-quality chemical and exposure related data from public documents via curation, quality assurance (QA), and visualization tools. With Factotum, there has been a significant focus on obtaining data on chemical composition of consumer products, functional role of chemicals within products and processes, and presence of chemicals on reported lists aligned with specific or general use. Ongoing efforts are underway to broaden the scope of the available data while ensuring data quality, through the addition of new data sources, extraction and curation of relevant product and chemical use information, curation of reported chemical identifiers to harmonized chemical identifiers, and implementation of QA workflows.

The initial version of EPA’s Chemical and Products Database (CPDat) was described in Dionisio *et al*.^11^ and was first made available online in 2017^13^; it will hereafter be referred to as CPDat v1.0. Since 2017, CPDat v2.0 (2017)^14^ and CPDat v3.0 (2021)^15^ have provided updated data to users of CPDat but have mostly maintained use categorization vocabularies from CPDat v1.0. The most current version, CPDat v4.0^16^, makes use of the new curation technologies implemented in Factotum to 1) more robustly link each chemical data record to its original data source, and 2) expand the quantity and quality of chemical and product data made publicly available. CPDat v4.0 also utilizes new controlled vocabularies, designed for exposure and chemical assessments, that form the basis of data search capabilities in a new public application for exploring CPDat data. With these updates, CPDat strives to improve its alignment with the FAIR principles^17^ of “findable, accessible, interoperable, and reusable” data.

Data contained within CPDat have played vital roles in parameterizing exposure models^18^, supporting government screening programs^8^, interpreting monitoring data^19^, identifying of compounds in non-targeted analysis of indoor/outdoor environmental^20–23^ or biological^24,25^ samples, and the prioritization of chemicals for further study on the basis of exposure potential^26–28^. New, better curated data will add new insights to studies such as these. In addition, the database can also inform the development of models that seek to fill gaps in exposure-relevant data such as Quantitative Structure Use Relationship (QSUR) models^29^.

## Methods

### The CPDat Pipeline: Making CPDat FAIR

**Fig. 1:**
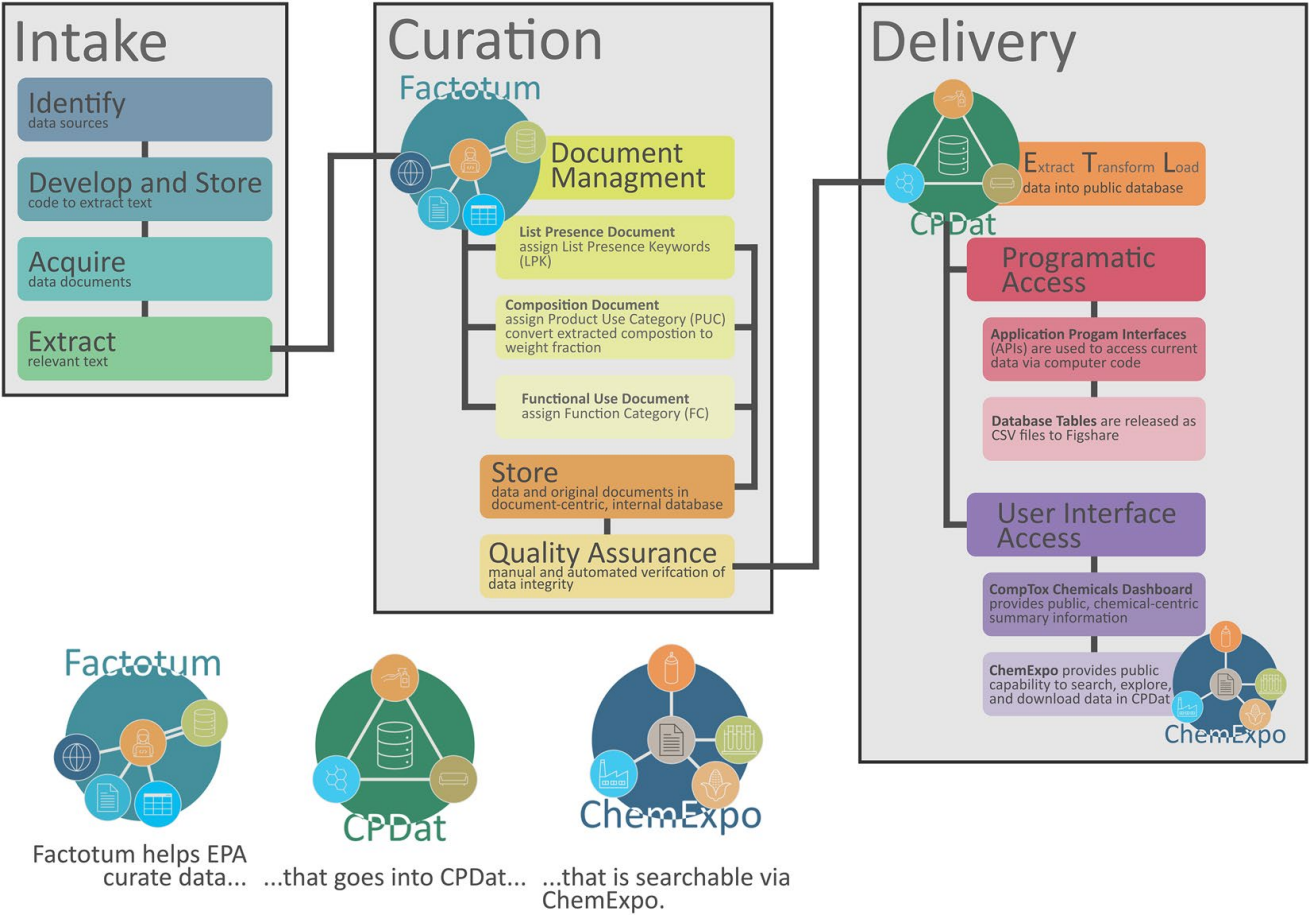
An overview of the CPDat Pipeline which includes intake of new data, curation of data records from extracted text, and delivery of curated data in multiple forms. The relationship between Factotum, CPDat, and ChemExpo is explained in the lower, right corner of the figure.

CPDat v1.0^13^ aggregated and harmonized several existing EPA databases or datasets^10,12,29^ and was primarily intended to store information about consumer uses of chemicals. Each of the data sources used to construct versions 1–3^13–15^ of CPDat were public at the time they were acquired. With each subsequent release of CPDat, new, publicly available data have been identified through either online searches or by users of CPDat alerting CPDat maintainers of new sources. Curating and incorporating these data manually or even in a semi-automated fashion can be time consuming and error prone. Alleviating these issues required developing a database and data pipeline (i.e., a set of data processing elements connected in series) that manage intake and processing of data documents, track original documents and their extracted information for quality assurance (QA) purposes, and deliver data so that it meets the FAIR data standard^17^. The implementation of these processes makes up the CPDat Pipeline (Fig. 1).

The CPDat Pipeline consists of three stages: Intake, Curation, and Delivery, with each of these stages being comprised of multiple tasks. The Intake stage includes the identification of publicly available, priority data sources by the CPDat team, acquisition of the identified data files and extraction of the relevant data. Data sources are prioritized by those most relevant to the use of chemicals and products in the United States (U.S.). Many data sources are associated with manufacturers or retailers whose products are in the U.S. market. However, some international data sources are included, particularly if those chemicals may represent some portion of the U.S. market. Relevant extracted data may include document metadata, chemical identifiers, product/brand name(s), and chemical use information. Acquisition and text extraction is handled either manually or via custom scripts (typically in the Python language). Scripts used to perform these tasks are stored in a GitHub repository (https://github.com/HumanExposure/data_mgmt_scripts) and linked to the original document (allowing for updates if needed during QA). Documents and extracted information are then passed to the Curation stage in the CPDat Pipeline, which is performed by persons trained in the use of EPA’s internal Factotum curation tool.

A CPDat *data document* consists of a raw data file, which could be a PDF, spreadsheet, HTML or other type of text-based file, and its extracted text. At the beginning of the Curation stage, *data documents* are matched to the correct *data document* type within Factotum. Currently, there are three *data document* types: *composition documents*, which provide information on the chemicals and their weight fraction in a product as well as how a product is intended to be used (e.g., spray vs. gel and/or home vs. outdoor use); *functional use documents*, which provide information on the specific role chemicals can serve in products or processes; and *list presence documents*, which provide information on the general use of chemicals in various settings. These *data document* types will be described further in subsequent sections. A human curator then uses the extracted data and metadata to assign appropriate terms from a controlled vocabulary to the data entries. The schema for each *data document* type has an associated controlled vocabulary; these vocabularies are also described in more detail in subsequent sections. Once a *data set* (all *data documents* of a certain type that are associated with a given data source) has had the relevant data and metadata extracted and had those data mapped to the appropriate vocabulary, it is then passed to the QA task, where a different human curator checks the vocabulary assignments and extracted text for accuracy against the raw data file. During this process, extracted chemical identifiers (e.g., chemical name, chemical abstract services registry number – CASRN) are registered in EPA’s Distributed Structure-Searchable Toxicity (DSSTox) database^30^ where substances are mapped to unique DSSTox Substance Identifiers (DTXSIDs)^31^; this chemical curation process is detailed in the Chemical Curation Section below. Once the QA task is approved, the curated and raw data are stored in a *data document*-centric database, accessible only to internal EPA users.

The Delivery stage entails performing extraction, transformation, and loading (ETL) of data from the document-centric database of Factotum to the product/use-centric database of CPDat, which is used to deliver data to the public.

### Data curation

#### CPDat curation using the Factotum system

EPA’s internal Factotum system is a data management platform into which all original files and any downstream curated data are entered, managed, and verified. Factotum implements structured tracking of data provenance (all records traceable to the original source file) and contains tools that support efficient data curation and quality assurance (QA) processes. Factotum and its underlying infrastructure are the origin of the reproducible extract–transform–load (ETL) CPDat Pipeline (Fig. 1) which is used to populate CPDat. Data entry is completed manually or in bulk (via upload of script-generated data) through Factotum’s user interface, which allows for curation of data and assignment of controlled vocabularies, via user-friendly, drop‐down menus and selection fields; the system also performs automated data validation checks.

Data curation completed through Factotum’s user interface is performed according to an overarching quality plan and documented standard operating procedures (SOPs). There are currently 18 SOPs that cover pipeline processes for different *data document* types and data records, including file uploading, manual and script-based data extraction (e.g., for *composition*, *list presence*, or *functional use documents*), data cleaning, creation of product data records and assignment to PUCs, chemical curation, and functional use curation. The SOPs are updated, at minimum, on an annual basis to reflect any changes in procedures, incorporation of new Factotum functionality, or addition of new curation or QA processes.

#### Chemical curation

Each unique chemical record associated with a *data document* has been assigned a chemical record ID, associated with a reported chemical name and CASRN, if available. Each of these individual chemical records are mapped to a DTXSID by the EPA’s chemical curation team using an automated workflow (these automated workflows also allow for manual checking and curation). Once mapped to a DTXSID, chemicals from CPDat then have access to preferred names, verified CASRNs, and chemical structures for that substance in DSSTox. Chemical records awaiting formal curation within DSSTox are assigned provisional DTXSID identifiers based on the official curation of other CPDat records with identical reported identifiers.

### Data documents

#### Composition documents

*Composition documents* are those that have been acquired from product manufacturers, trade associations, or retailers and which contain information on the ingredients in and/or quantitative composition of consumer products. These documents are typically in the form of (material) safety data sheets – (M)SDS, voluntary product ingredient disclosure documents, or other ingredient lists (such as those required by law for cosmetics or other personal care products). In some cases, these documents may also include reported functional use for ingredients in a product, though this is not always the case. It should be noted that *composition documents* containing function information differ from *functional use documents* described in the next section in that the data are associated with a specific chemical *and product*, as opposed to a chemical alone. Curation of these *composition documents* involves extraction of product names, reported product category from the original data source (if available), and chemical identifiers (e.g., chemical name or CASRN), composition information, and any functional use information (if available).

The consumer products represented in the *composition documents* are assigned to product use categories (PUCs)^32^. This controlled vocabulary was recently developed to explicitly aid in specifying the product type for use in exposure assessment and modeling; PUCs can be used to link specific products (and chemicals) to default information on consumer use patterns (e.g., prevalence and frequency of use), location of chemical release (e.g., area of the home or workplace), and exposure factors (e.g., area of skin likely to be exposed, percent remaining on skin after use). Many products have been provisionally assigned to a PUC using automated text recognition classification. These methods work by predicting a likely PUC for a product based on the brand name, product name and product manufacturer, allowing for the initial classification of thousands of products that would otherwise be awaiting time-intensive manual curation. A machine learning model trained on a manually curated subset of product-to-PUC assignments is used to make these predictions. While this internal model underwent rigorous evaluation and validation by exposure and machine learning subject matter experts at EPA, CPDat provides indicators of PUC assignment as manually assigned or modeled, allowing the user to subset the data by method of PUC assignment.

The PUCs used in CPDat v4.0 are an updated version of the 3-level PUC vocabulary described in detail in Isaacs *et al*.^32^ and first used in CPDat v3.0^15^; PUCs have been added and minor refinements have been made as new products were curated in CPDat v4.0. The CPDat v4.0 PUC vocabulary is a 4-level system expanded to cover additional product types, with each PUC now assigned to a PUC “kind”; PUC kinds include “formulation” (a product that is stored in a container, used up, and re-purchased; e.g., household cleaners, toothpastes), article (a durable good that is intended to be used long-term in an environment; e.g., building insulation, furniture), industrial/occupational (a product or material that is only intended to be used in a specific process or work environment; e.g., aircraft deicer, dry cleaning chemicals), and unknown. The remaining hierarchical levels are general category (“gen_cat”), product family (“prod_fam”) and product type (“prod_type”). As an example of PUC assignment: an oven cleaner product would be assigned the PUC “Formulation; Cleaning products and household care; appliance cleaner; oven cleaner”. The PUCs for articles are based on the Organisation for Economic Co-operation and Development’s (OECD) harmonized article categories^33^. Provisional industrial/occupational product PUCs were developed based on examination of the occupational products currently in the database and will be refined in the future.

The PUC hierarchy used in CPDat is evergreen: PUCs may be added, combined, or refined as necessary as new products are added to CPDat. Some PUCs are currently quite broad due to the limited number of products with which they are associated or because the products in CPDat are all likely to be used in the same way (e.g., the article PUC for “Toys and children’s products” contains various products that all result in dermal exposure during use). The PUC categories, and the specific products to which they are assigned, may change as new models or data become available for use in exposure modeling.

#### Functional use documents

*Functional use documents* are those obtained from product manufacturers or other organizations that describe the functional use, or role, chemicals play in products and/or processes. This information can be found on ingredient disclosure documents or in other documentation (e.g., sales materials) produced by manufacturers, distributors, or retailers and include information on the specific function that a chemical may serve in a product.

Whereas functional uses in Versions 1—3 of CPDat^13–15^ were restricted to a set of harmonized functional use categories described in detail in Phillips 2017 *et al*.^29^, functional uses in CPDat v4.0 have been curated to a standardized vocabulary recommended by the OECD^33^. CPDat v4.0 has combined the 117 harmonized technical functions published by the OECD (and recognized by international governing bodies, non-government organizations, and manufacturers) with an additional 13 functions of importance to the EPA, but not included in the OECD list (e.g., “pharmaceutical”, “depilatory”). This combination of functions is termed the Function Category (FC) vocabulary. In addition to the function categories themselves, the FC vocabulary also has a variable, FC “kind”, indicating the source of the FC as either OECD or EPA.

#### List presence documents

The *list presence documents* of chemicals on lists distributed by U.S. and international bodies allow for the inference of general chemical use. These lists may be found within public documents related to chemical use (e.g., lists of fragrances in consumer products), exposure potential, environmental occurrence, or regulatory purview. In EPA’s Chemical and Product Categories (CPCat) database^10^, a precursor to CPDat v1.0, *list presence documents* were used to assign chemicals to a set of defined terms covering broad categorizations of use such as commercial sectors in which a chemical was used as well as the types of products or roles a chemical could play in products. The unique set of terms defining a specific chemical list were called cassettes. Cassettes were meant to define the use context associated with the list of chemicals on the document. For example, a chemical could have been assigned the term “cosmetics” implying that the chemical was associated with chemicals in cosmetics products. However, if the chemical in that same document was also assigned terms of “banned” and “Europe”, it would indicate that the chemical was banned for cosmetic product use in European countries. These CPCat terms were maintained through CPDat v2.0^13,14^, but CPDat v3.0^14^ transitioned to an updated chemical List Presence Keywords (LPK) vocabulary originally described in Koval *et al*.^26^. In this vocabulary, *keywords* have replaced CPCat terms and *sets* have replaced CPCat cassettes.

The LPK vocabulary was developed to better align the assignment of broad use information with other CPDat data streams, namely functional use and consumer product composition data. Within CPDat v4.0 there are approximately 140 LPKs assigned to public lists. It is anticipated that the number of LPKs will grow as additional *list presence documents* are curated.

The transition from CPCat terms to the LPK vocabulary included four major refinements. The first refinement was to remove the keywords that are explicitly recognized as functional uses within the FC vocabulary. Similarly, keywords associated with a consumer product type have been aligned with the PUC vocabulary. For example, a public document may contain a list of chemicals that are used in lipsticks. Rather than using “lipstick” as the keyword, chemicals on this list would be assigned a keyword identical to the relevant PUC (i.e., Formulation; Personal Care; Make-up and related). In this way, CPDat v4.0 will still capture potential use for exposure scenarios even though there is not quantitative composition data associated with those chemicals (because they are on a *list presence document* rather than a *composition document*). As a third refinement, individual keywords which were pharmaceutical-related have been collapsed to a single “pharmaceutical” keyword. Finally, as a fourth refinement, individual LPKs have been assigned to an overarching higher-level LPK “kind” (similar to PUC and FC kinds), to aid in organization and summarization of the data. Currently, there are eleven LPK kinds (see Supplemental Information of Koval *et al*.^26^), but the organization of the LPK vocabulary allows for more kinds to be added, as needed. Just as in CPCat, keywords do not provide the full context of the use of a chemical obtained from a *list presence document*; rather the full set of keywords must be considered.

## Data Records

CPDat v4.0 is stored in a Figshare data repository and is available publicly at 10.23645/epacomptox.5352997^16^. The basis of all data records in CPDat are *data documents*. As mentioned previously, the three different types of *data documents* in CPDat (*composition documents*, *functional use documents*, and *list presence documents*) are described by a controlled vocabulary (PUCs, FCs, and LPKs, respectively) that helps organize documents that may be related in ways relevant for exposure or public health scientists (e.g. exposure pathway, route, population, chemical, etc.).

Information that is curated in Factotum and delivered to CPDat originates from publicly available data sources. A data source can have multiple data groups, which are groupings of documents of the same *data document* type. Table 1 shows the three different types of *data documents* available in CPDat v4.0 and the number of unique data sources and data groups for each type. Each data group can contain thousands of *data documents*.

**Table 1:** Counts of the number of unique data sources and data groups available in CPDat v4.0 for each type of data document type.

Data Document Type	Unique Data Sources	Unique Data Groups
Chemical presence list	30	302
Composition	48	102
Function	5	24

Originally, CPDat v1.0 was provided to the public as a MySQL file^13^. This file required users to have the means and knowledge to load a local version of a fairly large relational database onto their computer. Based on user feedback, this format limited access to CPDat as a typical user was unfamiliar with loading and querying a relational database. As a result, CPDat is now made available as a set of comma-separated value (CSV) files which are better known and usable by those in the public-health fields.

CPDat v4.0 includes 6 CSV files and one Excel file^16^. For each *data document* type there are two files: one file defines terms of the controlled vocabulary relevant to the data document type and another file provides relevant, curated data from data documents of that document type. The single excel file contains metadata for the other CPDat files. Each file is prepended by the string “cpdat_vX.X” where X.X denotes the version of CPDat. The CSV file names included in CPDat v4.0 are:

- **cpdat_v4.0_metadata.xlsx**: metadata related to the files and checks performed on those files in CPDat v4.0
- **cpdat_v4.0_fc_vocabulary.csv**: complete list of FCs used in CPDat v4.0
- **cpdat_v4.0_functional_use_data.csv**: all relevant data curated from *functional use documents* in CPDat v4.0
- **cpdat_v4.0_lpk_vocabulary.csv**: complete list of LPKs used in CPDat v4.0
- **cpdat_v4.0_list_presence_data.csv**: all relevant data curated from *list presence documents* in CPDat v4.0
- **cpdat_v4.0_puc_vocabulary.csv**: complete list of PUCs used in CPDat v4.0
- **cpdat_v4.0_product_composition_data.csv**: all relevant data curated from *composition documents* in CPDat v4.0

Each record in the files cpdat_v4.0_functional_use_data.csv, cpdat_v4.0_list_presence_data.csv, and cpdat_v4.0_product_composition_data.csv contains relevant metadata for accessing the original document (i.e., data source, group, and/or document URLs). While CPDat aims to capture public information related to the uses of chemicals in commerce and retain the provenance of that information, data sources may remove or move information so that the provided URL in CPDat no longer accesses the corresponding data. By providing URLs as well as download dates, CPDat does provide a way to use internet archives to access historical URLs.

The cpdat_v4.0_metadata.xlsx file contains 3 separate spreadsheets. The first sheet lists each of the files provided as part of the CPDat release and published references for the vocabularies used in CPDat. The second sheet lists the different columns used to describe the terminology for the CPDat data dictionary and the third sheet provides the data dictionary. This dictionary contains the name of each field, its definition, information on the type of data allowed in the field, any data checks that were performed on the field, and the name of the corresponding CSV file.

## Technical Validation

The original quality objectives for CPDat have not changed in that quality control and quality assurance efforts focus on accurately extracting and providing data that are in the raw data document. No effort is made on part of the CPDat team to follow-up with original data providers to ensure that the data in the document are, in fact, correct. Technical validation efforts are focused on ensuring that CPDat correctly delivers the data provided by the raw documents from online sources. In addition to the QA efforts that are built into the Factotum document management tool (described in the Methods section), automated checks and validations are performed on data during the ETL process. Data checks were implemented to ensure that if a range of weight fraction values are delivered via CPDat, then minimal or central values are not greater than maximal values.

Another data check was performed to find any instance of a null value that had been incorrectly ascribed to a text string and used as such in CPDat. During automated text extraction, if an expected value is left empty, then scripts will typically (and correctly) assign a NULL value to that field. However, if the raw document contains some varying form of “None”, “Not Provided”, “Null”, “N/A”, it can be challenging to catch each instance and convert it to the proper NULL value.

Finally, data frame schemata were implemented to ensure the expected values of any given field in the CPDat data files were not violated. Implementation and criteria for each data field are provided in the *cpdat_v4.0_metadata.xlsx* file included with CPDat v4.0.

## Usage Notes

In addition to the CPDat data files available on Figshare^16^, CPDat can be searched or visualized through other EPA Tools:

- CPDat data can be programmatically searched by a specific chemical identifier via APIs developed by EPA’s ORD. Usage of ORD’s Computational Toxicology and Exposure (CTX) APIs require an API key (instructions for obtaining a key can be found at https://api-ccte.epa.gov/docs/index.html).
- Summary data from CPDat can be obtained using EPA’s CompTox Chemicals Dashboard (https://comptox.epa.gov/dashboard).
- Detailed data from CPDat can be obtained using EPA’s Chemical Exposure Knowledgebase (ChemExpo; https://comptox.epa.gov/chemexpo). More information on using this resource, which may be a more approachable way to access CPDat for the typical public health user, is available in the ChemExpo User Guide^34^.

EPA’s ORD aims to provide releases to CPDat twice a year, provided availability of resources and significant new data. Identification and addition of new data sources (and the associated addition of new documents and data to CPDat) are driven by specific EPA exposure assessment needs, as well as an overarching goal to increase the product and chemical scope of CPDat.

CPDat will utilize a versioning system similar to that of Semantic Versioning used in software development (https://semver.org/). If only new data are being added to CPDat then addition of these data will constitute minor releases (i.e., CPDat v4.1). Should data curation issues be identified, a bug fix version may be released (i.e., CPDat v4.0.1). If substantial changes are made to the organization or vocabulary or if new types of data are added, this will result in a major release of CPDat (i.e., CPDat v5.0). Data infrastructures and processes are currently being built within EPA’s ORD to support the simultaneous and seamless releases of data versions across all applications (e.g., the CompTox chemicals Dashboard, the APIs, ChemExpo, etc.). However, the data version surfaced in any application is always reported.

## Data Availability

All scripts used to manage intake and extraction of new *data documents* into EPA’s Factotum tool can be found at https://github.com/HumanExposure/data_mgmt_scripts. The data files for CPDat v4.0 are located on Figshare^16^ (10.23645/epacomptox.5352997); note this will redirect to a specific EPA web address where the data release can be downloaded. Previous releases of CPDat can also be obtained from this site. Instructions for accessing CPDat data via the CTX APIs can be found at https://api-ccte.epa.gov/docs/index.html; note users must first request an API key before being able to use this interface. Currently, clients exist for these APIs in the R and Python programming languages. The R client is ctxR (https://cran.r-project.org/web/packages/ccdR/index.html). The Python client is ctx-python (https://pypi.org/project/ctx-python). Examples of using the R client are available via a GitHub Gist at https://gist.github.com/kaphillips/1237872438efae58f724e2155623520d. A separate Gist has been written to provide similar information for the Python client at https://gist.github.com/kaphillips/e40f2d8cf9ef13d7863678658a9770ce.
